# Supplementary material for: Mesoscale molecular architecture of the human striatum across cell types and lifespan
Source: bioRxiv. 2026 Mar 5:2026.03.04.709715. Preprint. [Version 1] doi: 10.64898/2026.03.04.709715 (PMC12991174; doi:10.64898/2026.03.04.709715)
Supplement: Supplement 3 [file media-3.pdf]

Puck	Age	Sex	Total Cells	Median nUMI All Cells	Median nUMI MSNs
s12	36	Male	96,335	10,433	56,076
s15	64	Male	98,292	11,919	55,136
s16	66	Male	99,057	11,424	68,791
s17	76	Female	64,844	12,123	66,366
s18	50	Female	69,597	9,480	68,028
s20	60	Male	46,066	12,410	20,219
s21	56	Male	127,834	13,074	78,506
s23	31	Male	151,068	19,912	68,513
s26	71	Male	90,927	14,196	22,811
s29	54	Male	98,415	10,967	42,556
s31	45	Male	82,729	21,547	84,728
s32	53	Male	137,759	7,841	49,782
s33	59	Male	79,824	10,681	65,570
s34	66	Male	83,502	11,946	48,429
s35	69	Male	122,537	10,209	54,116
s36	48	Male	93,376	9,176	45,324
s37	88	Female	108,240	10,507	47,873
s38	88	Female	94,774	12,896	38,439
s5	44	Male	133,467	20,475	125,554
