# Supplementary material for: Mesoscale molecular architecture of the human striatum across cell types and lifespan
Source: bioRxiv. 2026 Mar 5:2026.03.04.709715. Preprint. [Version 1] doi: 10.64898/2026.03.04.709715 (PMC12991174; doi:10.64898/2026.03.04.709715)
Supplement: 1 [file NIHPP2026.03.04.709715V1-supplement-1.pdf]

bioRxiv preprint doi: https://doi.org/10.64898/2026.03.04.709715; this version posted March 5, 2026. The copyright holder for this preprint (which was not certified by peer review) is the author/funder, who has granted bioRxiv a license to display the preprint in perpetuity. It is made available under aCC-BY 4.0 International license.

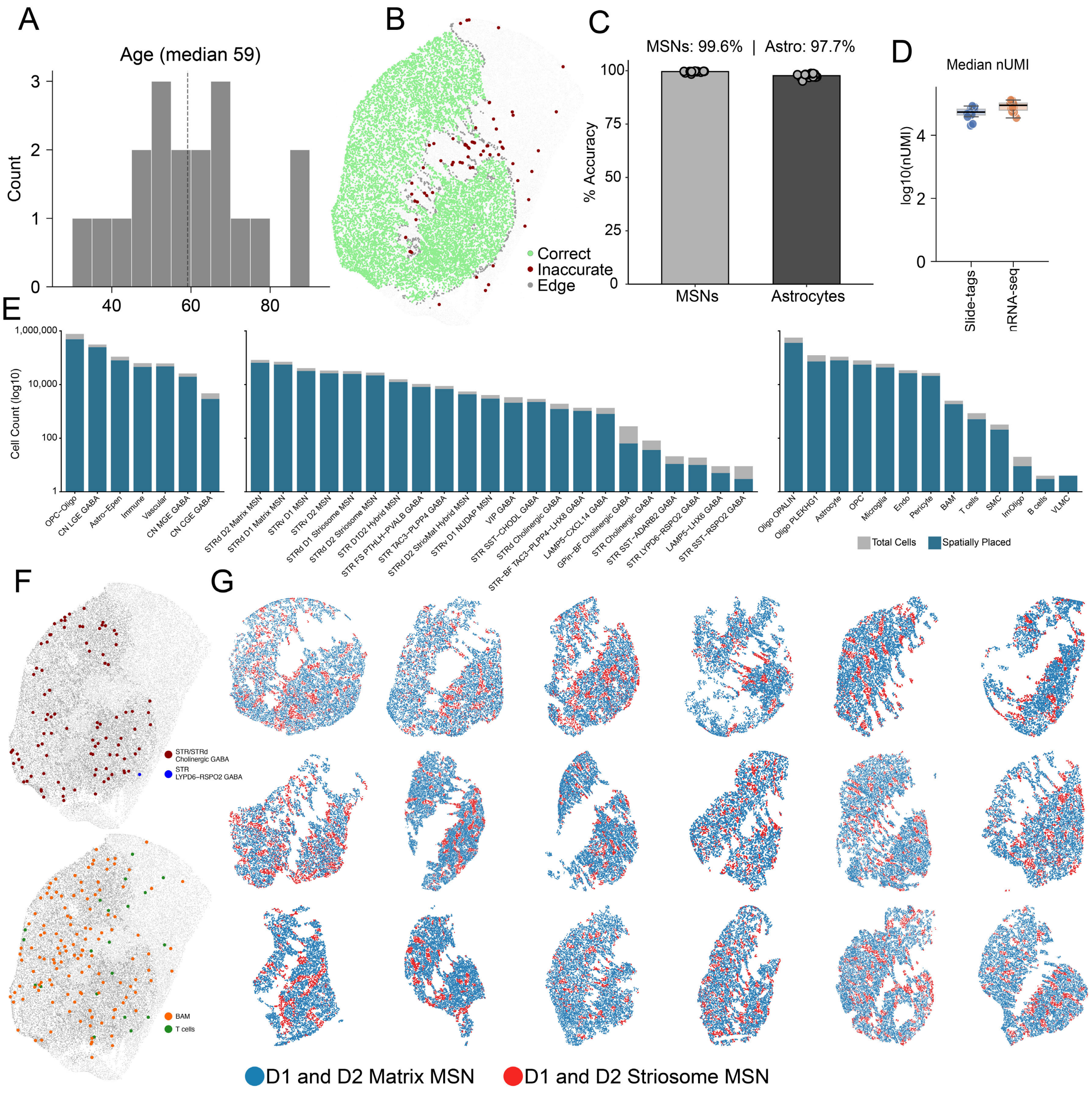

Figure S1. Cohort demographics, technical validation, and spatial cellular census of the Slide-tags human striatal atlas. A) Age distribution of the 19 postmortem donors profiled within the spatial cohort. **B)** Spatial mapping specificity evaluated in a representative donor. The medium spiny neuron (MSN) false-placement rate was quantified utilizing white matter tracts as an anatomical negative control (profiles inaccurately mapping to white matter are flagged in red). **C)** Quantification of spatial mapping accuracy for MSNs and astrocytes across the full 19-donor cohort, demonstrating highly specific spatial localization. **D)** Transcript capture sensitivity, displayed as the median unique molecular identifiers (nUMI) per MSN. Transcript recovery in the Slide-tags spatial cohort (n=19 donors) is comparable to a reference standard single-nucleus RNA-seq cohort (n=131 donors). **E)** Yield of total recovered and spatially localized nuclei across major cell classes (left), fine-grained neuronal subtypes (middle), and non-neuronal subtypes (right), annotated using a reference basal ganglia taxonomy[18]. **F)** Representative spatial localizations of selected neuronal (top) and non-neuronal (bottom) populations. **G)** Spatial distribution of the cardinal Matrix and Striosome MSN compartments across the remaining 18 donors. **H)** Inhomogeneous pair correlation (g_inhom(r)) evaluated against a background-fixed simulation envelope.

bioRxiv preprint doi: https://doi.org/10.64898/2026.03.04.709715; this version posted March 5, 2026. The copyright holder for this preprint (which was not certified by peer review) is the author/funder, who has granted bioRxiv a license to display the preprint in perpetuity. It is made available under aCC-BY 4.0 International license.

Simulation envelopes (gray ribbons) were generated from 199 Monte Carlo simulations of an inhomogeneous Poisson process under a background density estimated from all MSN cells; exceedance of the envelope indicates significant spatial clustering beyond tissue-level MSN density gradients.

bioRxiv preprint doi: https://doi.org/10.64898/2026.03.04.709715; this version posted March 5, 2026. The copyright holder for this preprint (which was not certified by peer review) is the author/funder, who has granted bioRxiv a license to display the preprint in perpetuity. It is made available under aCC-BY 4.0 International license.

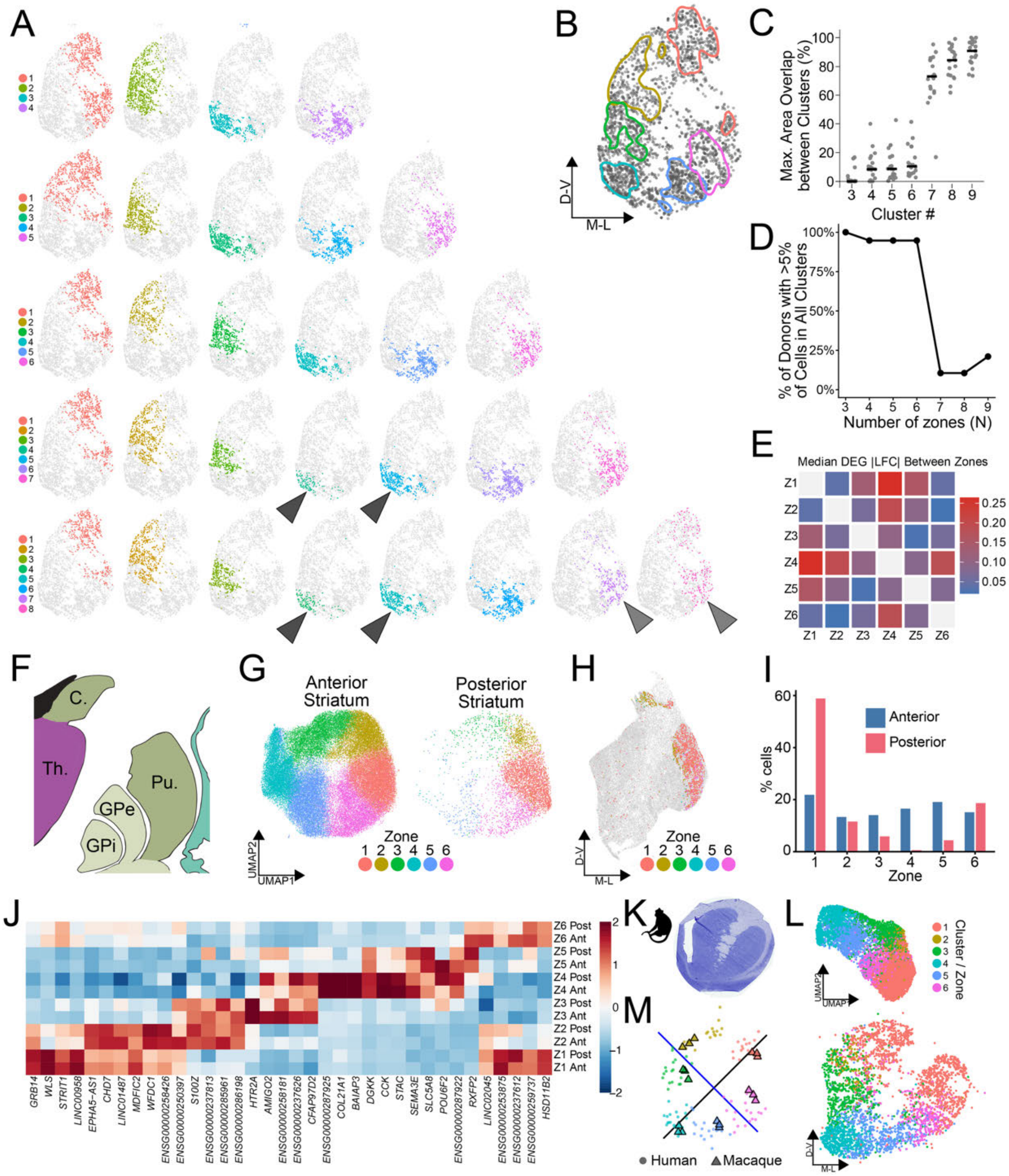

Figure S2. Additional spatial and cross-species validation of D1 Matrix zones. A) Spatial distribution of *k*=4 through *k*=8 clustering solutions in a representative donor (arrowheads: examples of high spatial overlap). **B)** KDE-derived contour maps of zonal territories. **C)** Maximum spatial overlap area between cluster pairs for each *k* solution. **D)** Minimum donor representation within any cluster as a function of cluster count**. E)** Median log-fold change magnitude of differentially expressed genes across all zone pairs. **F)** Anatomical diagram of the posterior striatum region profiled by Slide-tags (adapted from Ding et al., 2016[61]). **G)** Joint UMAP embedding of anterior (19 donors, left) and posterior (2 donors, right) D1 Matrix MSNs. **H)** Spatial distribution of posterior D1 Matrix MSNs in a representative donor. **I)** Percentage of D1 Matrix MSNs assigned to each zone in anterior versus posterior sections. **J)** Normalized expression of D1 Matrix zonal markers across anterior and posterior sections. **K)** Nissl stain of a coronal *Macaca fascicularis* striatum section adjacent to the Slide-tags assay. **L)** UMAP embedding (top) and corresponding spatial map (bottom) of macaque D1 Matrix neurons. **M)** Aligned spatial centroids for each zone across the 19 human donors (circles) and 3 adjacent macaque sections (triangles).

bioRxiv preprint doi: https://doi.org/10.64898/2026.03.04.709715; this version posted March 5, 2026. The copyright holder for this preprint (which was not certified by peer review) is the author/funder, who has granted bioRxiv a license to display the preprint in perpetuity. It is made available under aCC-BY 4.0 International license.

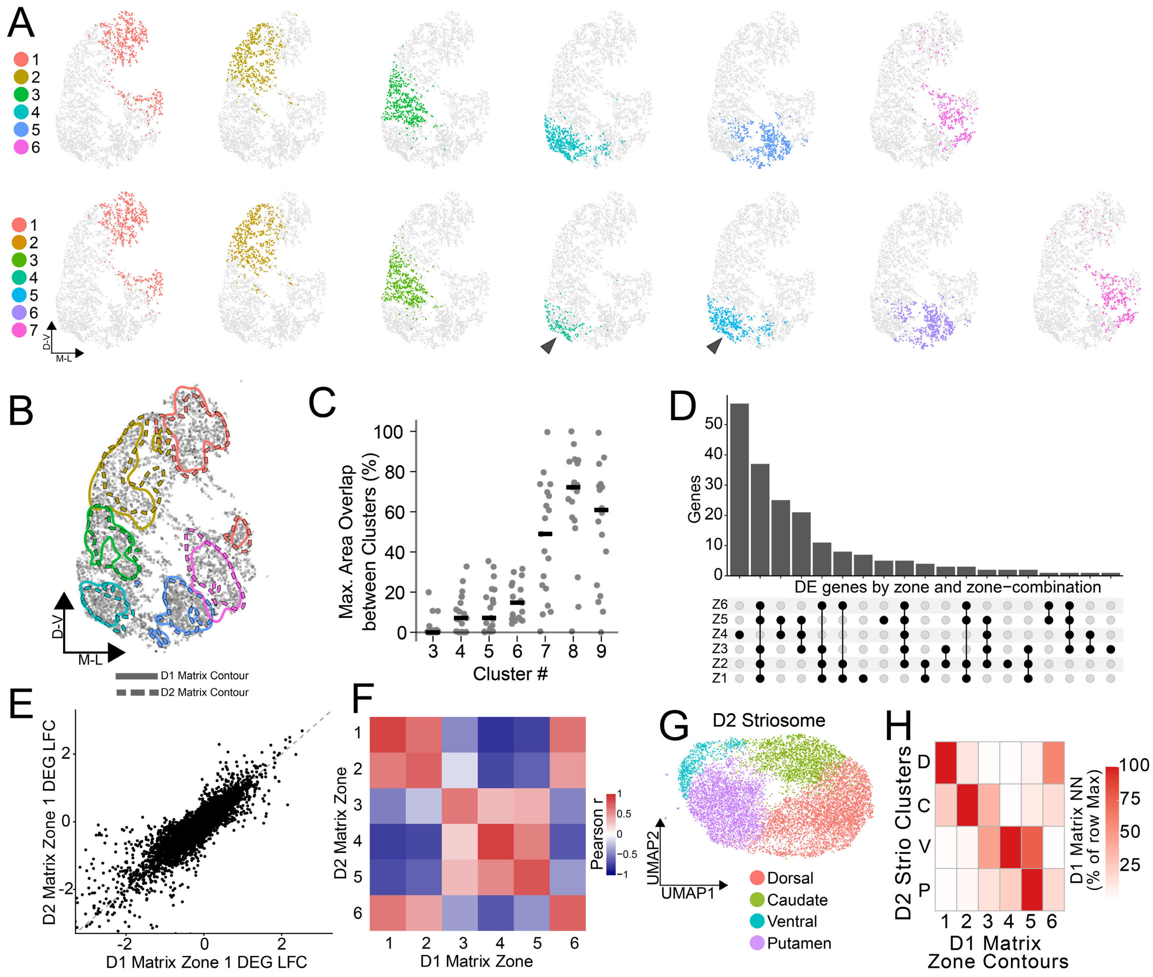

Figure S3. Extended spatial analysis of diverse MSN subtypes. A) Spatial distribution of *k*=6 (top) versus *k*=7 (bottom) clustering solutions for D2 Matrix MSNs in a representative donor. **B)** Contour maps comparing boundaries defined by D1 versus D2 Matrix MSNs. **C)** Maximum spatial overlap area between D2 Matrix cluster pairs across *k* solutions. **D)** UpSet plot detailing differentially expressed genes (DEGs) shared across single or combined D2 Matrix zones. **E)** Scatter plot comparing log-fold changes of Zone 1 marker genes between D1 and D2 Matrix MSNs. **F)** Cross-population correlation of zonal markers between D1 and D2 Matrix MSNs. **G)** UMAP embedding of D2 Striosome MSNs. **H)** Spatial contour overlap between D2 Striosome clusters (rows) and D1 Matrix zones (columns).

bioRxiv preprint doi: https://doi.org/10.64898/2026.03.04.709715; this version posted March 5, 2026. The copyright holder for this preprint (which was not certified by peer review) is the author/funder, who has granted bioRxiv a license to display the preprint in perpetuity. It is made available under aCC-BY 4.0 International license.

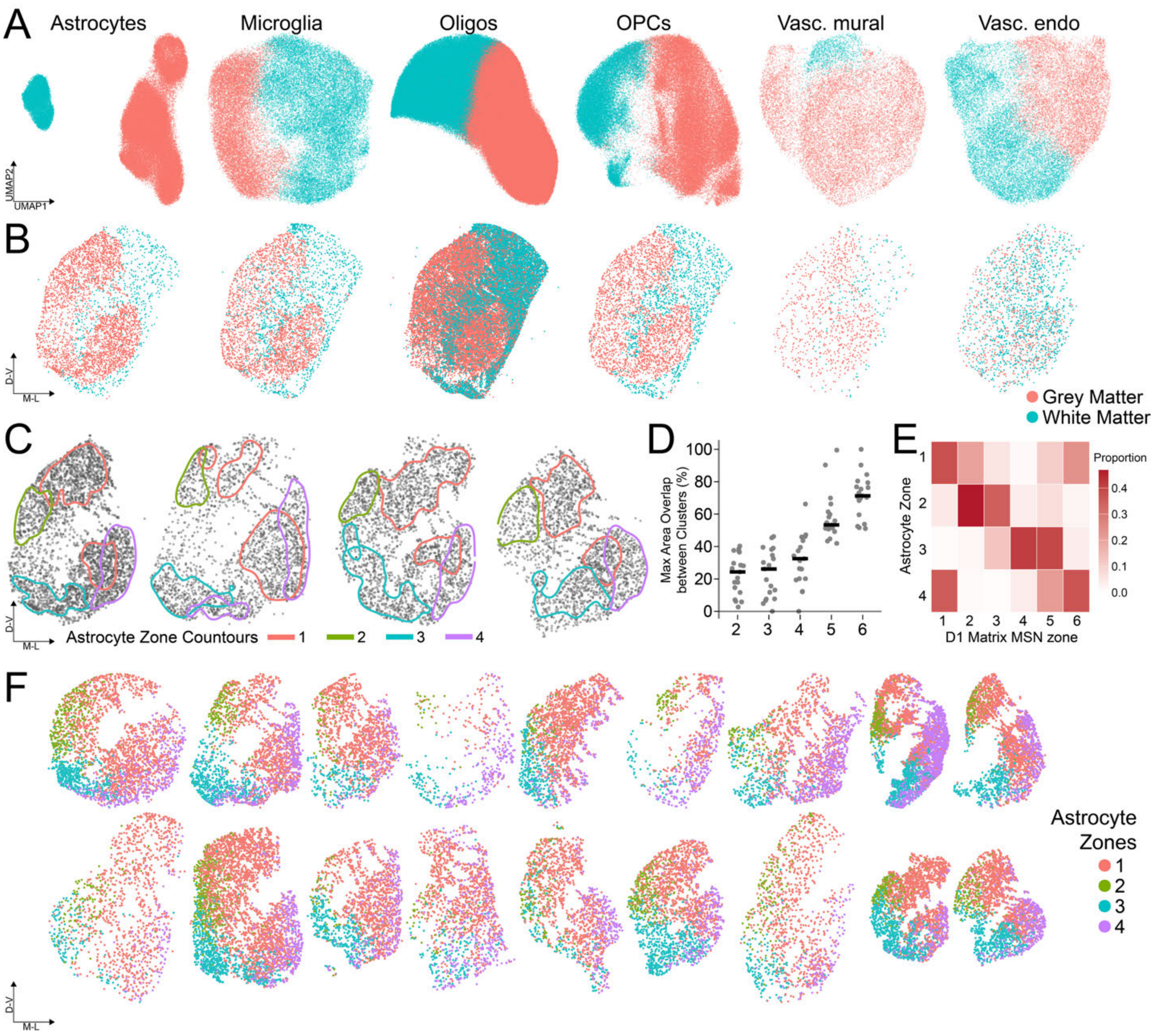

Figure S4. Spatial parcellation of striatal glia. A) UMAP embeddings and spatial **(B)** maps of glial populations distinguishing gray versus white matter identity. **C)** Contour maps of the four astrocyte zones identified via unsupervised clustering. **D)** Maximum spatial overlap area between astrocyte cluster pairs across *k* solutions. **E)** Spatial contour overlap between astrocyte clusters (rows) and D1 Matrix MSN zones (columns). **F)** Spatial distribution of zonated astrocytes across all 19 donors.

bioRxiv preprint doi: https://doi.org/10.64898/2026.03.04.709715; this version posted March 5, 2026. The copyright holder for this preprint (which was not certified by peer review) is the author/funder, who has granted bioRxiv a license to display the preprint in perpetuity. It is made available under aCC-BY 4.0 International license.

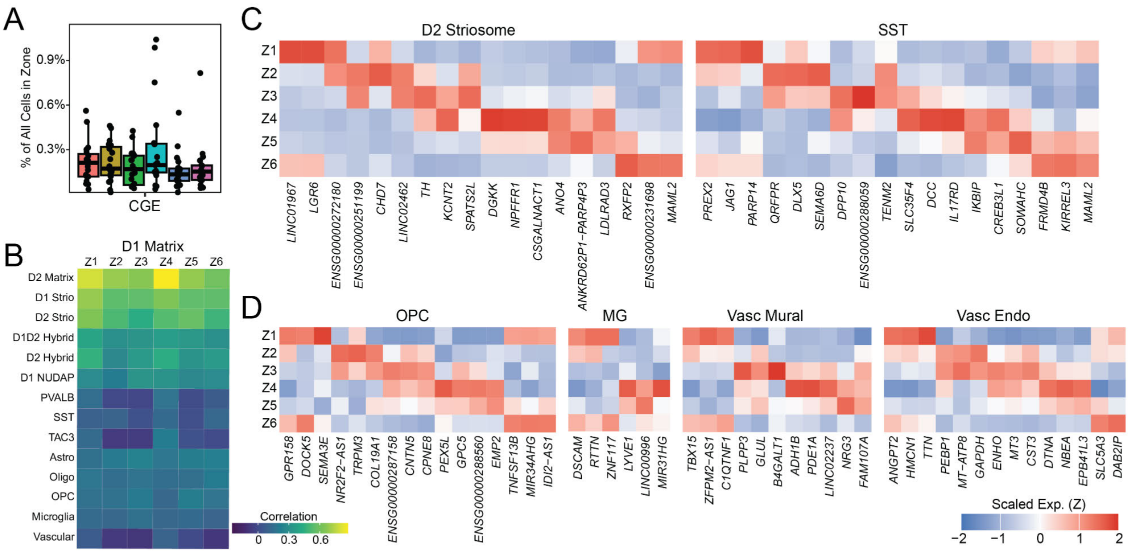

Figure S5. Striatal zones possess distinct cellular architectures and transcriptional specializations. A) CN CGE GABA neuron proportions for each sNN assigned zone. **B)** Cross-cell-type correlation of expression changes (log-fold change) for the union of zonal marker genes, comparing D1 Matrix MSNs to all other cell populations. **C-D)** Heatmap of normalized expression for representative zone-specific marker genes for **(C)** D2 Striosome MSNs, SST interneurons and **(D)** glial cells within each D1 Matrix-defined striatal zone.

bioRxiv preprint doi: https://doi.org/10.64898/2026.03.04.709715; this version posted March 5, 2026. The copyright holder for this preprint (which was not certified by peer review) is the author/funder, who has granted bioRxiv a license to display the preprint in perpetuity. It is made available under aCC-BY 4.0 International license.

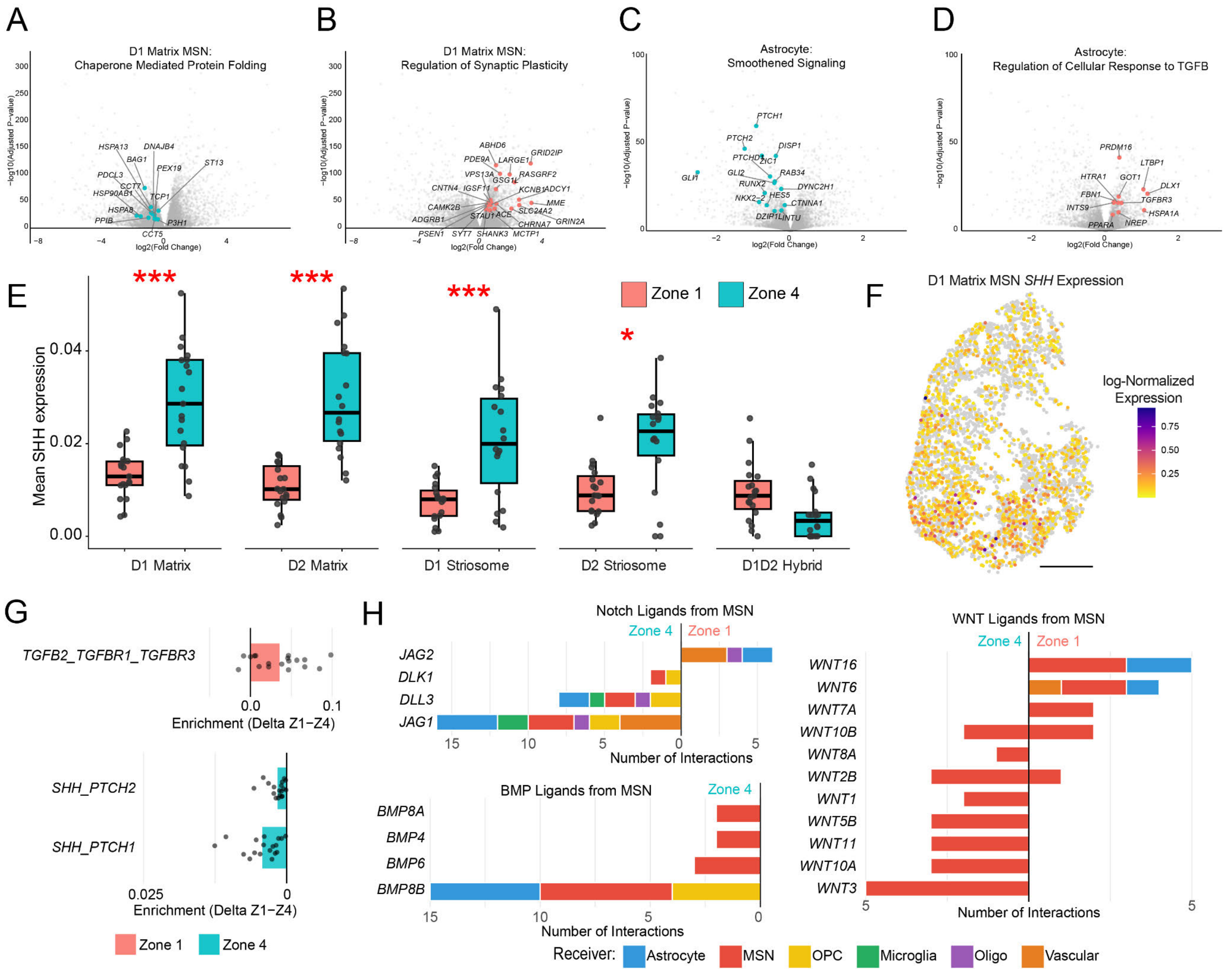

Figure S6. Zone-specific gene set enrichment in D1 Matrix MSNs and astrocytes. A,B) Volcano plots of DESeq2 results highlighting the top 30% of leading-edge genes from **(A)** Chaperone-Mediated Protein Folding and **(B)** Regulation of Synaptic Plasticity. **C,D)** Volcano plots of DESeq2 results highlighting the top 30% of leading-edge genes from **(C)** Smoothened Signaling and **(D)** Regulation of Cellular Response to TGF-β. **(E)** Mean expression levels of MSN *SHH* in Zones 1 and 4 across all donors (*p < 0.05, **p < 0.01, ***p < 0.001), and **(F)** corresponding spatial D1 Matrix MSN *SHH* expression map in a representative donor (scale bar = 0.5cm). **G)** Differential enrichment of *TGFB2* ligand interaction with the *TGFBR1* and *TGFBR3* complex in Astrocyte-to-Astrocyte signaling (top) and *SHH* ligand interactions in MSN-to-Astrocyte signaling (bottom). **H)** Interactions ranked by ligand for MSN-to-any cell type for Notch signaling (top left) and BMP signaling (bottom left) and Wnt (right) comparing the number of interactions between Zone 1 and Zone 4.

bioRxiv preprint doi: https://doi.org/10.64898/2026.03.04.709715; this version posted March 5, 2026. The copyright holder for this preprint (which was not certified by peer review) is the author/funder, who has granted bioRxiv a license to display the preprint in perpetuity. It is made available under aCC-BY 4.0 International license.

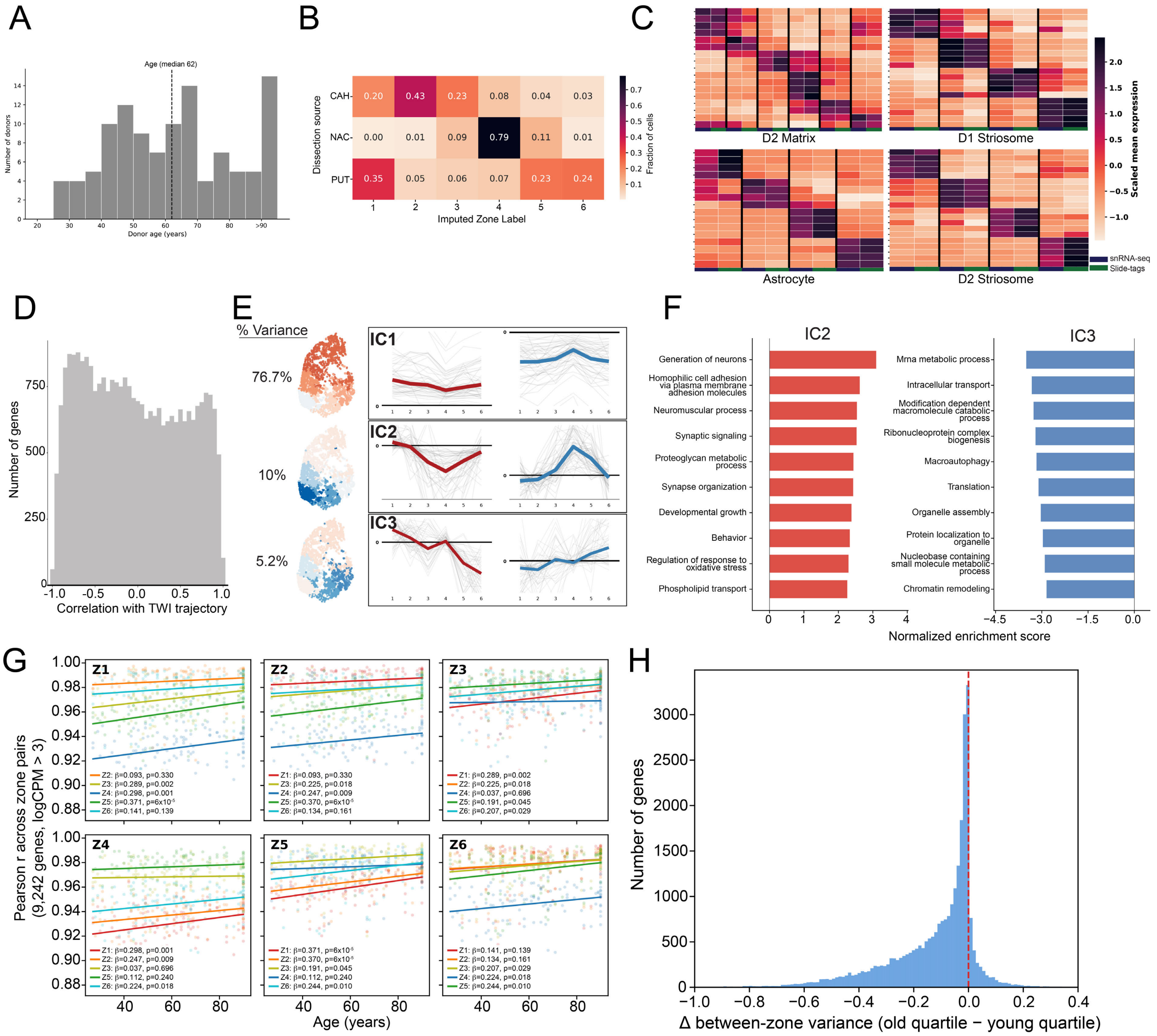

Figure S7. Validation of zonal imputation and expanded spatial aging analyses. A) Age distribution of the 131-donor snRNA-seq cohort. **B)** Confusion matrix comparing the macroscopic anatomical dissection region to the computationally imputed spatial zone. **C)** Normalized expression of canonical zonal markers across imputed D2 Matrix, D1 Striosome, D2 Striosome, and Astrocyte populations. **D)** Distribution of correlation coefficients between aging DEG logFC values and TWI across zones for all MSNs. **E)** Independent component analysis (ICA) of aging DEGs in D1 Matrix MSNs. Plots display the individual and average component loadings for the 50 highest- (red) and lowest-scoring (blue) genes. **F)** Gene Ontology (GO) pathway enrichment for Component 2 (left) and Component 3 (right). **G)** Age-dependent Pearson correlations of average gene expression (9,242 highly expressed genes, logCPM > 3) between all pairwise spatial zone combinations. **H)** Distribution of the change in between-zone expression variance (Δ variance) for D1 Matrix MSNs, comparing the oldest quartile (n = 30 donors, age > 75) to the youngest quartile (n = 28 donors, age < 49). 88.2% of genes (n=27,730) exhibit a negative Δ variance, indicating global, age-driven spatial compression. Statistical significance (P = 0.005) was assessed via 10,000 permutations shuffling donor age labels.
